# Phacoemulsification in a highly myopic, single-seeing, post-vitrectomized eye with an anterior chamber phakic intraocular lens: a surgical challenge

**DOI:** 10.31744/einstein_journal/2026RC2213

**Published:** 2026-04-30

**Authors:** Raquel Byel Kim, Diego Monteiro Verginassi, Carolina Engelbrecht, Cláudio Luiz Lottenberg

**Affiliations:** 1 Hospital Israelita Albert Einstein São Paulo SP Brazil Hospital Israelita Albert Einstein, São Paulo, SP, Brazil.

**Keywords:** Phacoemulsification, Myopia, Phakic intraocular lenses, Retinal detachment, Lens implantation, intraocular, Lenses, intraocular

## Abstract

We report a case of a highly myopic patient with a single functional eye, previously vitrectomized, and implanted with an anterior chamber phakic intraocular lens (Artisan^®^), who underwent phacoemulsification with phakic lens explantation and posterior chamber intraocular lens implantation. The case illustrates the surgical challenges and highlights the importance of individualized planning in patients with multiple prior interventions and at high risk of complications.

## INTRODUCTION

Anterior chamber phakic intraocular lenses (pIOLs) are intraocular devices implanted in the anterior chamber without removing the natural crystalline lens. They represent a viable alternative for the correction of moderate to high refractive errors, particularly in patients with contraindications to corneal refractive procedures.^(1,2)^

As these patients age, the natural crystalline lens may become opacified, necessitating cataract surgery (phacoemulsification). This procedure is technically challenging, as the anterior chamber lens must be removed before intraocular lens implantation in the capsular bag can be performed. Such cases often occur in individuals with high myopia, who are predisposed to retinal detachment, or in previously vitrectomized eyes due to prior retinal detachments. According to Han et al., each 6-diopter increase in myopia raises the risk of retinal detachment by approximately 7.2 fold.^(3)^

Among available anterior chamber phakic lenses, the Artisan^®^ lens, made of polymethylmethacrylate (PMMA) and fixed to the mid-peripheral iris stroma, stands out. Fixation in a relatively avascular area reduces the inflammatory response and ensures long-term tolerance. Its iris-claw fixation mechanism provides excellent stability and preserves accommodation.^(2,4)^

## CASE REPORT

A 54-year-old male patient with high myopia presented with a history of multiple ocular surgeries. In 1988, he developed retinal detachment in the right eye (OD) and underwent pars plana vitrectomy (PPV) without anatomical success, resulting in blindness in that eye (no light perception). In 2000, he developed retinal detachment in the left eye (OS), which was successfully treated with PPV and scleral buckle placement, achieving a best-corrected visual acuity (BCVA) of 20/60 (refraction: -16.00D).

In 2006, he developed intolerance to soft contact lenses and underwent implantation of an anterior chamber pIOL (Artisan^®^) in the left eye, with good postoperative outcomes.

**Figure 1:**
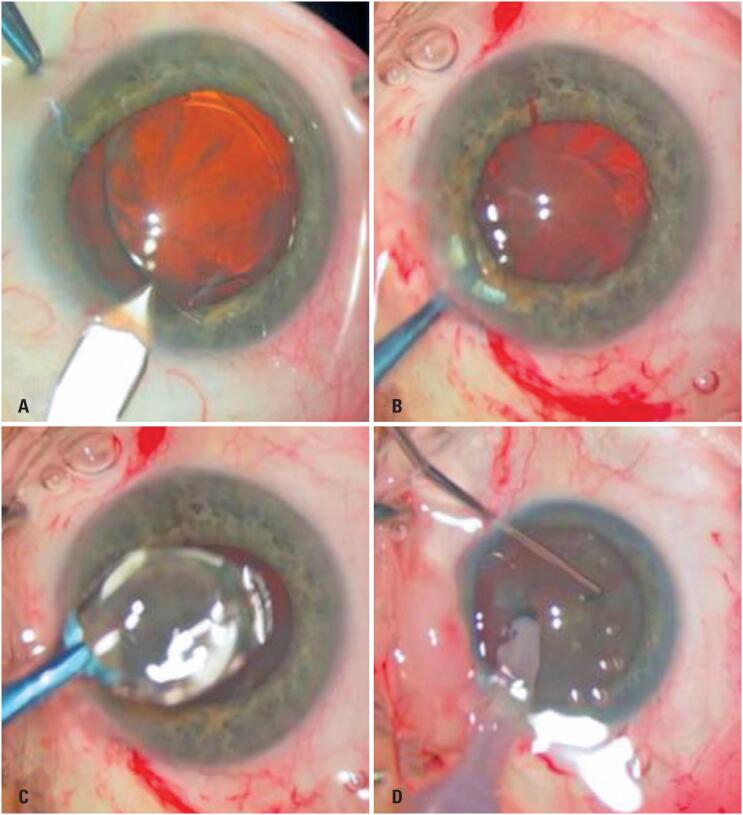
Intraoperative images of phacoemulsification with Artisan® lens explantation. (A) Main corneal incision; (B) Disenclavation of both haptics from the iris and grasping of the lens with specific forceps; (C) Lens explantation; (D) Corneal suturing (two stitches) and phacoemulsification with posterior chamber intraocular lens implantation in the capsular bag

In 2022, posterior subcapsular cataract (PSC 3+) developed in the left eye, reducing BCVA to 20/80. The patient underwent phacoemulsification with explantation of the Artisan^®^ lens and implantation of a posterior chamber intraocular lens (IOL) in the capsular bag during the same procedure. Postoperatively, BCVA improved to 20/60 (Figure 1).

**Figure 2:**
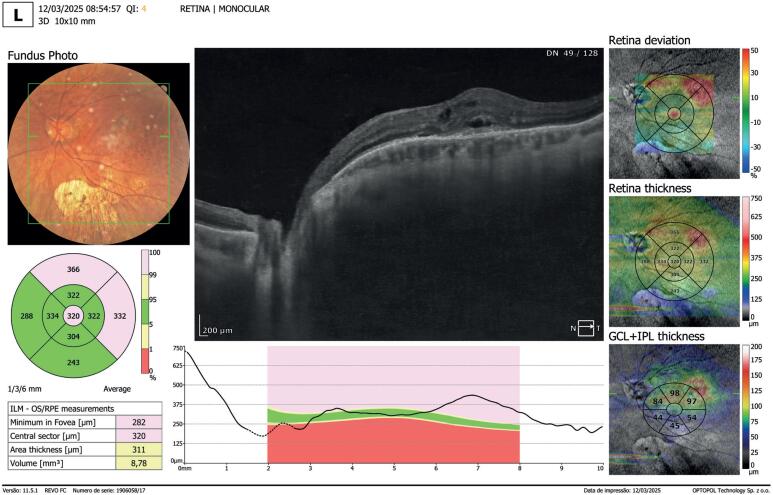
Macular optical coherence tomography and fundus photograph of the left eye showing intra- and subretinal fluid consistent with a myopic choroidal neovascular membrane

In 2023, he developed a new retinal detachment in the left eye and underwent a second PPV with C3F8 gas tamponade. Subsequently, he developed a neovascular membrane associated with myopic maculopathy in the same eye (Figure 2) and was treated with four intravitreal injections of ranibizumab (Lucentis^®^). The current BCVA in the left eye is 20/70.

This study was approved by the Research Ethics Committee of *Hospital Israelita Albert Einstein*, CAAE: 91778925.6.0000.0071; # 7.879.853.

## DISCUSSION

In patients with iris-fixated or anterior chamber phakic IOLs, the development of nuclear sclerosis does not appear to be directly related to lens implantation. However, once crystalline lens opacification leads to visual compromise, cataract surgery becomes necessary.^(5)^

In the present case, despite the elevated surgical risk due to multiple prior procedures and the fact that it was a single-seeing eye, the patient developed a PSC 3+ cataract, requiring phacoemulsification and Artisan^®^ explantation. The surgery was successfully performed in a single session, achieving satisfactory postoperative recovery and a BCVA of 20/60.

A retrospective observational study conducted in the Netherlands in 2009 evaluated 36 highly myopic eyes previously implanted with the Artisan^®^ lens that later underwent cataract surgery with lens explantation. The study demonstrated the safety of this approach, with no major complications such as retinal detachment, posterior capsule rupture, or permanent visual loss. Refractive predictability was favorable: 72.2% of eyes remained within ±1.00D of the target refraction, and 86.1% within ±2.00D. No patient lost lines of best-corrected visual acuity, confirming the stability and efficacy of the surgical technique.^(6)^

Vitrectomized eyes pose additional technical challenges due to the absence of vitreous support, making intraocular pressure stability more difficult to maintain during surgery. Nevertheless, the complication rate in procedures involving IOL extraction and iris-fixated IOL implantation reported by Ersöz et al. did not differ significantly between vitrectomized and non-vitrectomized eyes.^(7)^

The complexity of this case is further underscored by its rarity — multiple distinct surgical interventions in a single eye. Belin et al. retrospectively analyzed 2,115 eyes with rhegmatogenous retinal detachment and found an average of 1.68 procedures per eye over one year, with one extreme case requiring up to 10 interventions in the same eye during that period.^(8)^

## CONCLUSION

This case highlights the surgical management challenges in a highly myopic, single-seeing eye with a history of multiple ocular surgeries. The presence of an anterior chamber phakic intraocular lens requires careful preoperative planning when cataract surgery becomes necessary, particularly given the increased risk of complications such as retinal detachment.

Timely management of subsequent retinal detachments and prompt treatment of secondary complications (such as intravitreal injections for neovascular membranes) are critical for preserving visual function. Performing surgery before the cataract becomes advanced may reduce intraoperative risks and improve visual recovery, provided the decision balances potential benefits and risks.

Despite their complexity, surgical interventions in vitrectomized eyes can be safely performed with favorable outcomes when individualized planning, careful risk–benefit assessment, and appropriate surgical timing are prioritized.

## Data Availability

The underlying content is contained within the manuscript.
